# The impact of hypovolemia and PEEP on recirculation in venovenous ECMO: an experimental porcine model

**DOI:** 10.1186/s40635-024-00636-5

**Published:** 2024-06-01

**Authors:** Lars Prag Antonsen, Andreas Espinoza, Per Steinar Halvorsen, Itai Schalit, Harald Bergan, Didrik Lilja, Svein Aslak Landsverk

**Affiliations:** 1 Department of Anesthesia and Intensive Care, Rikshospitalet, Oslo University Hospital, Sognsvannsveien 20, 0372 Oslo, Norway; 2 Department of Anesthesia and Intensive Care, Østfold Hospital Trust, Kalnesveien 300, 1714 Grålum, Norway; 3 Department of Research, Østfold Hospital Trust, Kalnesveien 300, 1714 Grålum, Norway; 4 The Intervention Centre, Oslo University Hospital, Sognsvannsveien 20, 0372 Oslo, Norway; 5 Faculty of Medicine, University of Oslo, Problemveien 11, 0313 Oslo, Norway; 6 Department of Anesthesia and Intensive Care, Radiumhospitalet, Oslo University Hospital, Ullernchausseen 70, 0379 Oslo, Norway; 7 Department of Anesthesia and Intensive Care, Ullevaal Hospital, Oslo University Hospital, Kirkeveien 166, 0450 Oslo, Norway

**Keywords:** Extracorporeal membrane oxygenation, Recirculation, Intensive care, Animal model

## Abstract

**Background:**

Recirculation is a common problem in venovenous extracorporeal membrane oxygenation (VV ECMO) and may limit the effect of ECMO treatment due to less efficient blood oxygenation or unfavorable ECMO and ventilator settings. The impact of hypovolemia and positive end expiratory pressure (PEEP) on recirculation is unclear and poorly described in guidelines, despite clinical importance. The aim of this study was to investigate how hypovolemia, autotransfusion and PEEP affect recirculation in comparison to ECMO cannula distance and circuit flow.

**Methods:**

In anesthetized and mechanically ventilated pigs (*n* = 6) on VV ECMO, we measured recirculation fraction (RF), changes in recirculation fraction (∆RF), hemodynamics and ECMO circuit pressures during alterations in PEEP (5 cmH_2_O vs 15 cmH_2_O), ECMO flow (3.5 L/min vs 5.0 L/min), cannula distance (10–14 cm vs 20–26 cm intravascular distance), hypovolemia (1000 mL blood loss) and autotransfusion (1000 mL blood transfusion).

**Results:**

Recirculation increased during hypovolemia (median ∆RF 43%), high PEEP (∆RF 28% and 12% with long and short cannula distance, respectively), high ECMO flow (∆RF 49% and 28% with long and short cannula distance, respectively) and with short cannula distance (∆RF 16%). Recirculation decreased after autotransfusion (∆RF − 45%).

**Conclusions:**

In the present animal study, hypovolemia, PEEP and autotransfusion were important determinants of recirculation. The alterations were comparable to other well-known factors, such as ECMO circuit flow and intravascular cannula distance. Interestingly, hypovolemia increased recirculation without significant change in ECMO drainage pressure, whereas high PEEP increased recirculation with less negative ECMO drainage pressure. Autotransfusion decreased recirculation. The findings are interesting for clinical studies.

**Supplementary Information:**

The online version contains supplementary material available at 10.1186/s40635-024-00636-5.

## Introduction

Recirculation during venovenous extracorporeal membrane oxygenation (VV ECMO) is a common phenomenon in which oxygenated blood from the return cannula is withdrawn by the drainage cannula without entering the patient’s systemic circulation. Recirculation might limit the effect of ECMO treatment due to less efficient patient oxygenation and/or unfavorable ECMO and ventilator settings, including excessive ECMO flow and high airway pressures [1]. Thus, avoiding unnecessary recirculation is important, even when patient oxygenation is adequate.

ECMO is the most advanced treatment option for critical heart- and lung failure in patients not responding to conventional intensive care. Safe and effective treatment presents difficult tradeoffs. Transfusion and fluid resuscitation may be necessary to control bleeding and hypovolemia [2], while positive fluid balance may increase mortality [3–5]. Positive end-expiratory pressure (PEEP) contributes to lung protection in severe respiratory failure but increases intrathoracic pressure and may negatively influence patient hemodynamics [6]. High ECMO circuit flow may improve systemic arterial oxygen saturation [7], but have negative side effects such as hemolysis, inflammation, coagulation, cavitation and vasoconstriction [8, 9]. Optimal cannula positioning facilitates adequate blood flow at low ECMO pump speed. In contrast, malpositioned cannulas may increase recirculation, and cause blood vessel trauma, bleeding and blood cell damage—all potentially leading to end organ damage [10–14].

Several factors affect recirculation. These include cannula position, distance, size and configuration, as well as ECMO circuit flow, heart rate (HR) and native cardiac output (CO) [1, 10, 11, 15]. The impact of hypovolemia and PEEP on recirculation is still unclear and poorly described in guidelines, despite clinical importance in patients on VV ECMO [3, 4].

The aim of this animal study was to investigate and quantify how hypovolemia, PEEP and autotransfusion affect recirculation compared to ECMO circuit flow and intravascular cannula distance.

## Methods

Six healthy Noroc pigs of either sex were included in this experimental animal study. Median weight was 61.5 kg (58–67 kg). The study protocol was approved by the Norwegian National Animal Research Authority (trial registration number 24306 and 28798) and was performed in accordance with European legislation [16]. A porcine model was chosen because of the similarities to human cardiac anatomy and physiology [17]. We performed eight pilot experiments to plan the study. The experimental protocol was performed in ten animals. Recirculation measurements were obtained in six pigs. Out of ten pigs, one pig died before measurements were made. In two pigs, measurements were not available due to blood gas analyzer malfunction. In one pig, the presumed “S_cv_O_2_” was mistakenly drawn from the PA catheter, due to human error. Randomization and blinding were not applicable in this study protocol.

The pigs were subjected to fasting overnight with free access to water in an animal research facility, and premedicated by intramuscular injection of 30 mL ketamine 50 mg/mL (~ 25 mg/kg), 4 mL azaperone 40 mg/mL (~ 2.5 mg/kg) and 1 mL atropine 1 mg/mL (~ 15 μg/kg). Anesthesia was maintained with pentobarbital 4 mg/kg/h, morphine 2 mg/kg/h and midazolam 0.15 mg/kg/h. Ringer’s acetate solution was infused at 10 mL/kg/h until the start of the interventions. Total blood volume was estimated to ~ 60 mL/kg (~ 3600 mL).

Electrocardiography (ECG), peripheral oxygen saturation (S_p_O_2_), bladder temperature, invasive arterial pressure, central venous pressure and pressures from the inflow and outflow limbs of the ECMO circuit were obtained using standard bedside monitors (Life Scope® Monitor, Nihon Kohden, Japan). Pulmonary artery (PA) pressure was measured with a pulmonary artery catheter (Swan-Ganz CCOmbo, Edwards Lifesciences, Irvine, CA). All pressures were zeroed to atmospheric pressure and calibrated according to manufacturer’s specifications. Recordings were digitally transferred to Labchart Pro software (AD Instruments Ltd., Auckland, New Zealand). Blood gas samples, including central venous oxygen saturation (S_cv_O_2_) were analyzed bedside with a standard blood gas analyzer (ABL90 Flex, Radiometer Medical ApS, Denmark).

After anesthesia induction, the animals were mechanically ventilated via a tracheostomy with tidal volume 8–10 mL/kg, respiratory rate (RR) 16–18/min and inspired oxygen fraction (F_i_O_2_) 0.5. After ECMO initiation and before the start of the experimental protocol, tidal volume was reduced to 4–5 mL/kg. Respiratory rate and F_i_O_2_ was not changed.

**Fig. 1:**
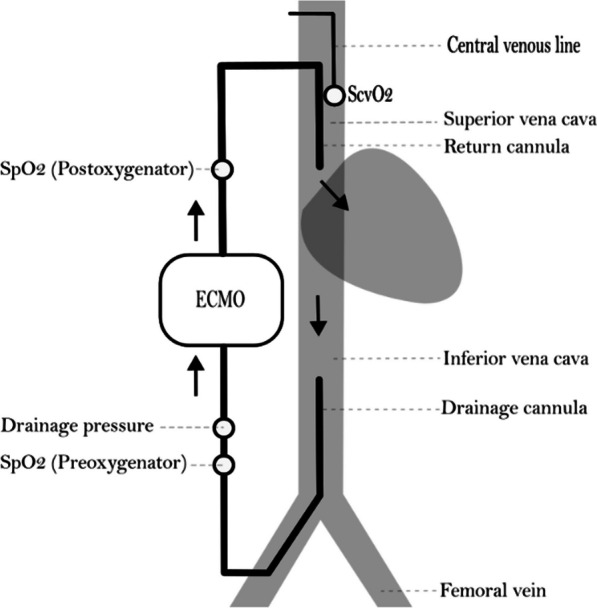
ECMO configuration. White circles indicate sampling sites of S_preoxy_O_2_, S_postoxy_O_2_, S_cv_O_2_ and ECMO drainage pressure

A PA catheter and a central venous line (CVL) were inserted via the internal jugular veins. The ECMO return cannula (17 French, Bio-Medicus™, Medtronic Inc., Fridley, United States) was inserted in the right external jugular vein with the cannula tip in the right atrium. The drainage cannula (23 French, Maquet Cardiopulmonary GmbH, Rastatt, Germany) was inserted via the right femoral vein with the cannula tip in the inferior vena cava (IVC) and adjusted to long cannula tip distance (20–26 cm) or short cannula tip distance (10–14 cm) according to the protocol below. The cannulas were inserted percutaneously ultrasound-guided using Seldinger’s technique during fluoroscopy to ensure proper positioning (Fig. 1).

**Fig. 2:**
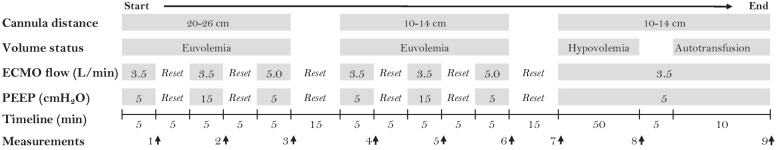
Timeline of interventions. Hemodynamics and recirculation fraction (RF) were measured with different combinations of fluid status, PEEP, ECMO flow and cannula position (arrows)

To prevent ECMO circuit clotting, a bolus of intravenous heparin 2 mg/kg followed by an infusion of 0.5 mg/kg/h to obtain an activated clotting time (ACT) of 180–200 s was given. The ECMO circuit was run in the femoro-atrial direction by a standard ECMO machine and oxygenator (Cardiohelp/HLS Set Advanced 7.0, Maquet Cardiopulmonary GmbH, Rastatt, Germany). Pump speed and circuit flow were adjusted according to the protocol in Fig. 2.

### Estimating recirculation

The recirculation fraction (RF) is the fraction of total ECMO blood-flow that circulates directly from the return cannula back into the drainage cannula without entering the patient’s systemic circulation. We used the CVL method which calculates RF based on S_cv_O_2_, preoxygenator oxygen saturation (S_preoxy_O_2_) and postoxygenator oxygen saturation (S_postoxy_O_2_) as follows: RF (%) = (S_preoxy_O_2_–S_cv_O_2_)/(S_postoxy_O_2_–S_cv_O_2_) × 100. (1) We estimated recirculation with combinations of euvolemia, hypovolemia (1000 mL, ~ 28% of total blood volume), PEEP, ECMO circuit flow and cannula distance according to the timeline in Fig. 2.

### Statistical analysis

Data were analyzed in MATLAB (R2022b, The MathWorks Inc, Natick, MA) and comparisons were made by the Wilcoxon signed rank test with *p* ≤ 0.05 considered significant.

### Experimental protocol

After the anesthetic and surgical preparations, we initiated ECMO treatment and measured RF according to the timeline in Fig. 2. The experimental protocol started with intravascular cannula distance 20–26 cm, ECMO flow 3.5 L/min and PEEP 5 cmH_2_O. After a 5-min adaptation phase, we obtained the first set of hemodynamic data, blood samples and RF estimates (Measurement 1). Then followed a 5-min stabilization phase before the next intervention.

Next, we applied PEEP 15 cmH_2_O and allowed a 5-min adaptation phase, before obtaining a second set of measurements (Measurement 2). The intervention was completed after resetting PEEP to 5 cmH_2_O and a 5-min stabilization phase.

Thereafter, we increased ECMO flow to 5.0 L/min and allowed a 5-min adaptation phase, before obtaining a third set of measurements (Measurement 3). The intervention was completed after resetting ECMO flow to 3.5 L/min and a 15-min stabilization phase.

We then repositioned the ECMO cannula and performed the same interventions (PEEP and flow) with intravascular cannula distance 10–14 cm (Measurements 4–6).

Hypovolemia was induced by gradually withdrawing 1000 mL blood in 50 min. (Measurements 7 and 8). Finally, autotransfusion was performed by infusing 1000 mL blood in 10 min. Measurements were obtained after a 5-min adaptation phase (Measurement 9).

## Results

**Fig. 3:**
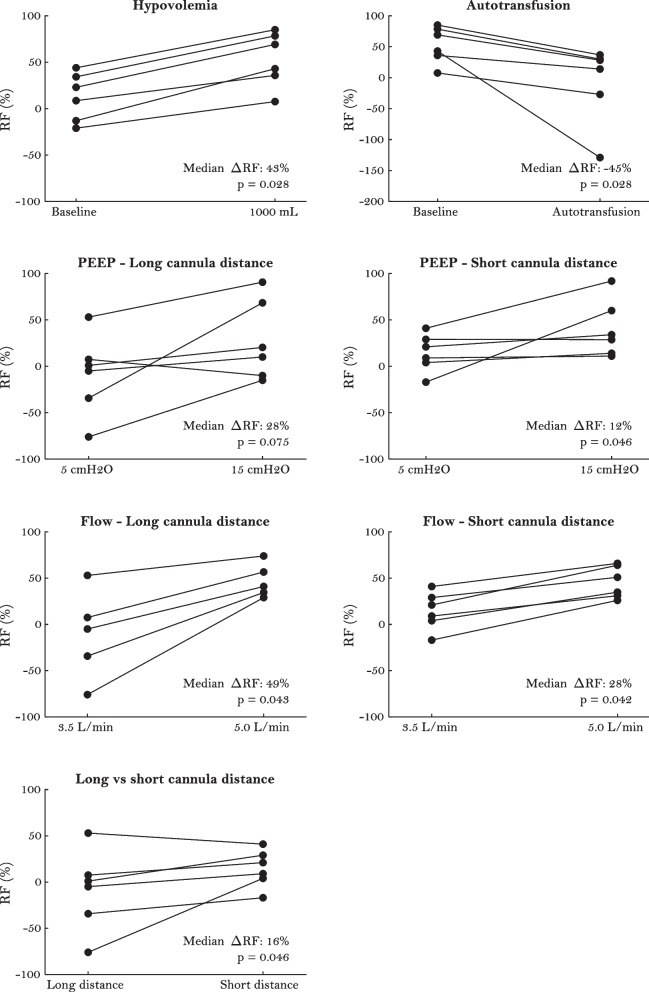
Impact of hypovolemia, autotransfusion, PEEP, ECMO flow and cannula distance on recirculation. One pig was excluded from analysis in subplot “Flow—Long cannula distance” due to technical malfunction

*Hypovolemia* induced a median increase in recirculation fraction (∆RF) of 43% (Fig. 3). HR increased, while mean arterial pressure (MAP), central venous pressure (CVP), mean pulmonary arterial pressure (MPAP) and arterial oxygen partial pressure (P_a_O_2_) decreased. ECMO drainage pressure did not change significantly, and there was no cannula chattering (Table 1).

**Table 1:** Impact of hypovolemia, autotransfusion, PEEP, ECMO flow and cannula distance on hemodynamics

	Hypovolemia			Autotransfusion		
	Baseline	Hypovolemia	*p* value	Hypovolemia	Autotransfusion	*p* value
HR (bpm)	108 (56 to 140)	161 (145 to 202)	0.028*	161 (145 to 202)	88 (58 to 135)	0.028*
MAP (mmHg)	84 (69 to 106)	43 (36 to 94)	0.027*	43 (36 to 94)	89 (65 to 102)	0.028*
CVP (mmHg)	7 (− 2 to 11)	4 (− 3 to 8)	0.027*	4 (− 3 to 8)	7 (0 to 10)	0.027*
MPAP (mmHg)	22 (15 to 28)	16 (13 to 21)	0.027*	16 (13 to 21)	23 (15 to 24)	0.028*
ECMO_prepump_ (mmHg)	− 51 (− 71 to − 34)	− 55 (− 81 to − 36)	0.112	− 55 (− 81 to − 36)	− 48 (− 70 to − 30)	0.027*
Preoxygenator S_p_O_2_ (%)	61 (56 to 73)	72 (58 to 88)	0.116	72 (58 to 88)	57 (49 to 67)	0.028*
S_cv_O_2_ (%)	54 (41 to 66)	33 (21 to 57)	0.028*	33 (21 to 57)	51 (38 to 78)	0.028*
P_a_O_2_ (kPa)	32 (27 to 35)	29 (25 to 32)	0.028*	29 (25 to 32)	29 (27 to 34)	0.463

	PEEP—Long cannula distance			PEEP—Short cannula distance		
	PEEP 5 cmH_2_O	PEEP 15 cmH_2_O	*p* value	PEEP 5 cmH_2_O	PEEP 15 cmH_2_O	*p* value
HR (bpm)	101 (60 to 136)	123 (90 to 144)	0.027*	91 (53 to 150)	138 (105 to 155)	0.046*
MAP (mmHg)	82 (62 to 94)	59 (33 to 84)	0.028*	82 (69 to 118)	64 (44 to 98)	0.042*
CVP (mmHg)	6 (− 1 to 10)	8 (2 to 14)	0.027*	6 (− 1 to 11)	8 (2 to 14)	0.027*
MPAP (mmHg)	20 (15 to 23)	25 (20 to 28)	0.027*	20 (17 to 23)	25 (19 to 30)	0.043*
ECMO_prepump_ (mmHg)	− 55 (− 75 to − 33)	− 51 (− 69 to − 31)	0.026*	− 55 (− 72 to − 32)	− 52 (− 62 to − 30)	0.027*
Preoxygenator S_p_O_2_ (%)	55 (52 to 67)	63 (56 to 92)	0.043*	62 (54 to 72)	65 (58 to 93)	0.345
S_cv_O_2_ (%)	58 (36 to 76)	53 (18 to 66)	0.173	54 (45 to 61)	46 (19 to 58)	0.072
P_a_O_2_ (kPa)	31 (27 to 34)	28 (24 to 30)	0.039*	31 (26 to 35)	30 (20 to 32)	0.042*

	ECMO Flow—Long cannula distance			ECMO Flow—Short cannula distance		
	Flow 3.5 L/min	Flow 5 L/min	*p* value	Flow 3.5 L/min	Flow 5 L/min	*p* value
HR (bpm)	118 (60 to 136)	137 (80 to 160)	0.080	91 (53 to 150)	122 (55 to 178)	0.043*
MAP (mmHg)	80 (62 to 94)	86 (56 to 100)	0.686	81 (69 to 118)	86 (51 to 105)	0.600
CVP (mmHg)	6 (− 1 to 7)	6 (0 to 8)	0.059	6 (− 1 to 11)	7 (− 2 to 12)	0.480
MPAP (mmHg)	19 (15 to 23)	20 (15 to 21)	1.000	20 (17 to 23)	20 (16 to 24)	0.892
ECMO_prepump_ (mmHg)	− 54 (− 65 to − 33)	− 118 (− 143 to − 101)	0.043*	− 53 (− 72 to − 32)	− 117 (− 158 to − 101)	0.027*
Preoxygenator S_p_O_2_ (%)	53 (52 to 67)	76 (70 to 87)	0.043*	60 (54 to 72)	77 (64 to 84)	0.042*
S_cv_O_2_ (%)	57 (36 to 76)	54 (44 to 69)	0.465	54 (45 to 61)	54 (44 to 64)	0.400
P_a_O_2_ (kPa)	31 (27 to 34)	31 (29 to 34)	0.450	31 (26 to 35)	32 (28 to 35)	0.273

	Cannula distance			Pre ECMO cannulation	
	Long distance	Short distance	*p* value		Baseline
HR (bpm)	101 (60 to 136)	91 (53 to 150)	0.753	HR (bpm)	72 (58 to 142)
MAP (mmHg)	82 (62 to 94)	82 (69 to 118)	0.345	MAP (mmHg)	80 (66 to 96)
CVP (mmHg)	6 (− 1 to 10)	6 (− 1 to 11)	0.564	CVP (mmHg)	10 (3 to 11)
MPAP (mmHg)	20 (15 to 23)	20 (17 to 23)	0.317	MPAP (mmHg)	20 (18 to 24)
ECMO_prepump_ (mmHg)	− 55 (− 75 to − 33)	− 55 (− 72 to − 32)	0.344	ECMO_prepump_ (mmHg)	NA
Preoxygenator S_p_O_2_ (%)	55 (52 to 67)	62 (54 to 72)	0.027*	Preoxygenator S_p_O_2_ (%)	NA
S_cv_O_2_ (%)	58 (36 to 76)	54 (45 to 61)	0.248	S_cv_O_2_ (%)	73 (59 to 83)
P_a_O_2_ (kPa)	31 (27 to 34)	31 (26 to 35)	0.332	P_a_O_2_ (kPa)	30 (18 to 48)

HR: heart rate, MAP: mean arterial pressure, CVP: central venous pressure, MPAP: mean pulmonary artery pressure, ECMO_prepump_: ECMO drainage pressure, S_cv_O_2_: central venous oxygen saturation, P_a_O_2:_ arterial oxygen partial pressure. Significant changes are marked *. One pig was excluded from analysis in table “Flow—Long cannula distance” due to technical malfunction

*Autotransfusion* of 1000 mL blood in 10 min caused a median ∆RF of − 45% (Fig. 3). HR decreased, while MAP, CVP and MPAP increased. P_a_O_2_ did not change significantly. ECMO drainage pressure became significantly less negative (Table 1).

*High PEEP* (15 cmH_2_O) caused a median ∆RF of 28% with a long intravascular cannula distance, and a median ∆RF of 12% with a short cannula distance (Fig. 3). There were large variations, and the changes with long cannula distance were not significant. In both positions, high PEEP increased HR, CVP and MPAP, while MAP and P_a_O_2_ decreased. ECMO drainage pressure became less negative, and there was no cannula chattering (Table 1).

*High ECMO flow* (5.0 L/min) caused a median ∆RF of 49% with a long cannula distance, and a median ∆RF of 28% with a short cannula distance (Fig. 3). There were large variations. HR increased with short cannula distance, but not significantly with long cannula distance. There were no significant changes in MAP, CVP, MPAP or P_a_O_2_. ECMO drainage pressure became increasingly negative, but there was no cannula chattering (Table 1).

*Repositioning the cannula* from 20–26 cm to 10–14 cm intravascular distance caused a median ∆RF of 16% (Fig. 3). There were large variations, and only minor changes in MAP, HR, CVP, MPAP, P_a_O_2_ and ECMO drainage pressure (Tables 1, 2).

**Table 2:** Ventilator settings

		Long cannula distance			Short cannula distance				
	Pre ECMO	Baseline	15 cmH_2_O	5.0 L/min	Baseline	15 cmH_2_O	5.0 L/min	Hypovolemia	Autotransfusion
RR (pr/min)	16 (16–18)	16 (16–18)	16 (16–18)	16 (16–18)	16 (16–18)	16 (16–18)	16 (16–18)	16 (16–18)	16 (16–18)
TV (mL/kg)	8–10	4–5	4–5	4–5	4–5	4–5	4–5	4–5	4–5
F_i_O_2_ (%)	50	50	50	50	50	50	50	50	50
PEEP (cmH_2_O)	5	5	15	5	5	15	5	5	5

RR: respiratory rate, TV: tidal volume, F_i_O_2_: inspired oxygen fraction, PEEP: positive end-expiratory pressure

## Discussion

In this study, hypovolemia, PEEP and autotransfusion were important determinants of recirculation. The alterations were comparable to other well-known factors, such as ECMO circuit flow and intravascular cannula distance. There were large variations in both recirculation and hemodynamics, supporting previous studies that report difficulties in measuring recirculation correctly (1). All findings support the notions that 1) recirculation is a multifactorial phenomenon and might increase with and without changes in drainage pressure, as well as with or without hemodynamic changes and 2) that recirculation is ultimately determined by how circulatory and respiratory physiology, as well as ECMO circuit configuration, affect blood supply to the ECMO drainage cannula [18].

### Hypovolemia and autotransfusion

A likely explanation for recirculation during hypovolemia is that a lower circulating blood volume leads to IVC collapsibility, a partial occlusion of the drainage cannula side holes and drainage through the cannula tip only. The result is a functionally shorter distance between the return cannula and the drainage cannula, ultimately leading to increased recirculation. It follows from the Poiseuille equation on flow resistance that with cannula sideholes open, blood mainly drains through the proximal side holes, not the cannula tip. Thus, the draining point within the IVC may vary several centimeters solely due to IVC collapsibility [19]. In addition, hypovolemia and reduced IVC inflow probably cause more blood to be directed through the ECMO drainage cannula without passing through the patient's circulatory system and thus increases RF even with all cannula sideholes open [18, 20]. Our study suggests that recirculation increases during hypovolemia and, importantly, may do so without increasingly negative drainage pressure or cannula chattering.

### PEEP

A likely explanation for the large variations in recirculation during high PEEP is related to heart–lung interactions, as cyclic variations in intrathoracic and intra-abdominal pressure lead to alterations in venous supply to the IVC drainage cannula throughout the respiratory cycle [1]. These cyclic variations highlight that accurate estimation of recirculation requires a representative blood sample obtained throughout the whole respiratory cycle. This source of error may partially explain the lack of consistency in recirculation measurements in general. Specifically, high PEEP leads to increased CVP followed by an increase in IVC diameter and improved drainage from all cannula holes. Occasionally, the variations were visually apparent as alternating blood color in the drainage cannula [21]. Our study suggests that recirculation increases during high PEEP and, in contrast to recirculation during hypovolemia, may do so with less negative drainage pressure.

### ECMO circuit flow

Recirculation increased during high ECMO circuit flow. Increasing ECMO pump speed leads to increasingly negative drainage pressure, IVC wall collapsibility, occlusion of the drainage cannula sideholes, drainage through the cannula tip only and likely to a functionally shorter intravascular cannula distance as described above. In addition, increased outflow from the return cannula is directed towards the drainage cannula. P_a_O_2_ was unaltered, indicating that oxygen delivery remained adequate despite increased recirculation [12].

### Cannula distance

Intravascular cannula position, distance and configuration are important determinants of recirculation, but there are no clear recommendations on the ideal position or separation between the drainage cannula and the return cannula [1, 10, 18, 20, 22]. Several cannulation strategies have been proposed, including reverse ECMO flow direction (atrio-femoral or femoro-femoral), x-configurations [23], dual lumen cannula, cannulas with expanding net baskets that prevent vessel walls from collapsing around the cannula [19], right ventricular cannula [24] and pulmonary artery return cannula. All have their benefits and disadvantages, but none have so far proven superior efficiency [25].

In clinical practice, maximizing the distance between the two cannulas is widely used to reduce the possibility of recirculation [1]. However, withdrawing the drainage cannula into a smaller blood vessel may lead to cannula sidehole occlusion and a functionally shorter distance between the cannulas as described above, paradoxically not reducing recirculation.

In addition, a drainage cannula in the lower IVC mainly draws blood from the lower body, allowing poorly oxygenated blood from the SVC to enter the heart without circulating through the ECMO circuit. In the upper IVC position, blood from the SVC, IVC, and hepatic vein is probably more uniformly drained. Thus, the supply of venous blood at the drainage site appears to be more important than cannula tip proximity per se, and altogether these mechanisms may limit how far the drainage cannula may be withdrawn [18, 22]. Our data suggest that retracting the cannula too far may not reduce recirculation as effectively as expected.

### Limitations

The most important limitation in this study is the CVL method to estimate recirculation. The CVL method is clinically applicable, but not always accurate as true S_cv_O_2_ is difficult to obtain in patients on VV ECMO [26]. First, because oxygen saturation in the SVC and the IVC vary substantially. Second, because the blood sample from the CVL may be partially mixed with oxygenated blood from the ECMO return cannula. Hence, S_cv_O_2_ obtained from the CVL may be falsely elevated, leading the RF equation to be negative if S_cv_O_2_ is higher than the S_preoxy_O_2_. However, the trends we report are consistent and indicate methodological robustness regardless of absolute values and measuring method.

There are several other ways to measure the RF. A practical approach is to compare S_preoxy_O_2_ and S_p_O_2_. Increasing S_preoxy_O_2_ and concomitantly decreasing S_p_O_2_ are early indicators of recirculation [1]. Other methods include using systemic arterial blood gas analysis to indicate or exclude significant recirculation [27, 28]. These methods, however, do not provide quantification of the RF. Quantitative RF measuring methods are based on S_cv_O_2_, thermodilution, lithium dilution, ultrasound dilution, oxygen content and/or sweep gas adjustments [1, 10, 11, 29].

Other limitations include the small number of animals and possible interactions between the interventions. Future studies should confirm our findings with other measuring methods and investigate different cannula types, sizes and configurations.

## Conclusion

In the present animal study, hypovolemia, PEEP and autotransfusion were important determinants of recirculation. The alterations were comparable to other well-known factors, such as ECMO circuit flow and intravascular cannula distance. Interestingly, hypovolemia increased recirculation without significant change in ECMO drainage pressure and without cannula chattering. High PEEP increased recirculation with less negative ECMO drainage pressure. Autotransfusion decreased recirculation. The findings are interesting for clinical studies.

### Supplementary Information

Supplementary Material 1. Video showing alternating blood color in the ECMO drainage cannula.

## Data Availability

The data sets generated and analyzed during the current study are available from the corresponding author on reasonable request.

## Abbreviations

ACT: Activated clotting time
CO: Cardiac output
CVL: Central venous line
CVP: Central venous pressure
ECG: Electrocardiography
F_i_O_2_: Inspired oxygen fraction
HR: Heart rate
IVC: Inferior vena cava
MAP: Mean arterial pressure
MPAP: Mean pulmonary arterial pressure
PA: Pulmonary artery
P_a_O_2_: Arterial oxygen partial pressure
PEEP: Positive end expiratory pressure
RF: Recirculation fraction
RR: Respiratory rate
S_cv_O_2_: Central venous oxygen saturation
S_postoxy_O_2_: Postoxygenator oxygen saturation
S_p_O_2_: Peripheral oxygen saturation
S_preoxy_O_2_: Preoxygenator oxygen saturation
SVC: Superior vena cava
VV ECMO: Venovenous extracorporeal membrane oxygenation
∆RF: Changes in recirculation fraction
